# Trunnion fracture of femoral prosthesis following a large metal-on-metal uncemented total hip arthroplasty: a case report

**DOI:** 10.1186/s42836-020-00055-3

**Published:** 2020-10-28

**Authors:** Krishna Kiran Eachempati, Chandra Sekhar Dannana, Sunil Apsingi, Vinay Kishore Ponnala, Giridhar Boyapati, Apurve Parameswaran

**Affiliations:** Department of Orthopaedics, Medicover (formerly MaxCure) Hospital, Opposite Cyber Gateway, Ibis Hotel lane, Hi-Tech City, Hyderabad, Madhapur, Telangana State 500081 India

**Keywords:** Trunnion fracture, Metal on metal, Revision hip arthroplasty, Trunnionosis, Uncemented hip arthroplasty

## Abstract

**Background:**

Trunnion fracture of the femoral prosthesis is an extremely rare complication following Total Hip Arthroplasty (THA). There are very few reports in literature on trunnion fracture. All previously reported cases are of prostheses with smaller heads, unlike the large metal-on-metal articulation in our case, which is unique.

**Case presentation:**

A 29-year-old male patient with juvenile idiopathic arthropathy presented to us with a fracture of the trunnion of his left THA, with no history of trauma. He had undergone staged bilateral THA for avascular necrosis of the hips 10 years ago (with AML-A stem and large metal-on-metal articulation bilaterally). We revised the THA and exchanged both femoral and acetabular components. He had a good functional outcome at 3 years’ follow up.

**Conclusion:**

The primary reason for the trunnion fracture in our case was the faulty stem design of the AML [with a small (9/10) taper, and a large head], causing excessive bending moment at the trunnion, worsening the cantilever effect and leading to subsequent cyclic fatigue failure. Whilst gross trunnion failure (GTF) with dissociation of the head from the taper is commonly reported, trunnion fracture *per se* is a rare and devastating complication. The AML-A stem has since been recalled by the company and there is a need to constantly monitor these patients for potential trunnion-related complications.

## Introduction

Trunnion fracture of the femoral stem is a rare complication following total hip arthroplasty (THA). It has been reported in a few cases of uncemented THA with small heads, and either a metal-on-polyethylene or ceramic-on-polyethylene articulation [1, 2]. Recently, fractures and dissociations of bimodular neck components have also been reported [3, 4]. In this report, we demonstrate an extremely rare case of a trunnion fracture following a large uncemented metal-on-metal THA. To our knowledge, it is the first such report in literature.

## Case report

A 29-year-old male patient (height 1.72 m; weight 74 kg; Body Mass Index 25) with a past history of juvenile idiopathic arthritis and chronic steroid use, underwent staged bilateral THA for arthritis secondary to avascular necrosis 10 years ago (2006). The postoperative course was uneventful and a good functional outcome was achieved. He could resume his normal day-to-day and recreational activities. He was regularly followed up for clinical assessment and radiographic evaluation. His blood levels of cobalt and chromium were serially monitored and were within acceptable limits. His radiographs prior to the primary surgery were unavailable.

**Fig. 1:**
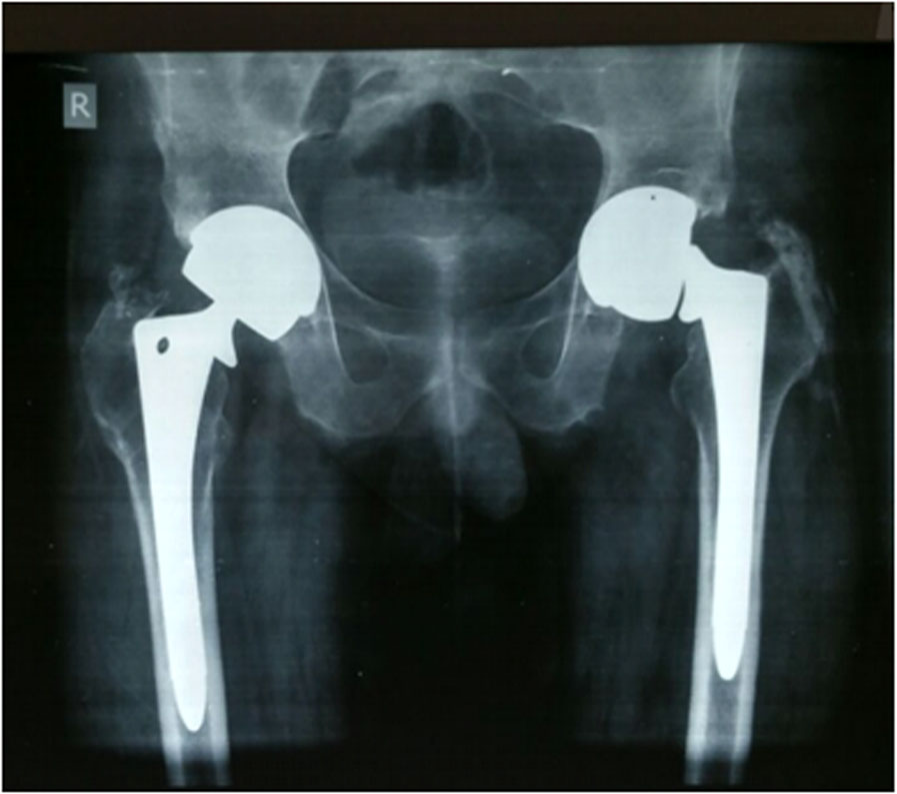
A plain radiograph at the time of presentation, showing bilateral large uncemented metal-on-metal total hip arthroplasty, with trunnion fracture on the left hip

He presented to us with a sudden onset of pain around his left hip while walking on the street, without any history of trauma. He was unable to bear weight following the onset of the pain. Radiographs obtained at the time of his presentation revealed a fracture at the level of the trunnion on the left side, with well-fixed femoral and acetabular components (Fig. 1). Based on the information obtained from his prior reports, the prostheses used during the primary surgery included an uncemented articular surface replacement (ASR) acetabular component with an outer diameter of 54 mm (DePuy Johnson and Johnson, India), a fully porous coated uncemented size14 anatomic medullary locking (AML) A plus femoral stem (DePuy International, Leeds, England), and a cobalt-chrome alloy head of 47 mm diameter with + 6 mm neck offset. His inflammatory markers, C-reactive protein, erythrocyte sedimentation rate, and leucocyte counts were within the normal range.

**Fig. 2:**
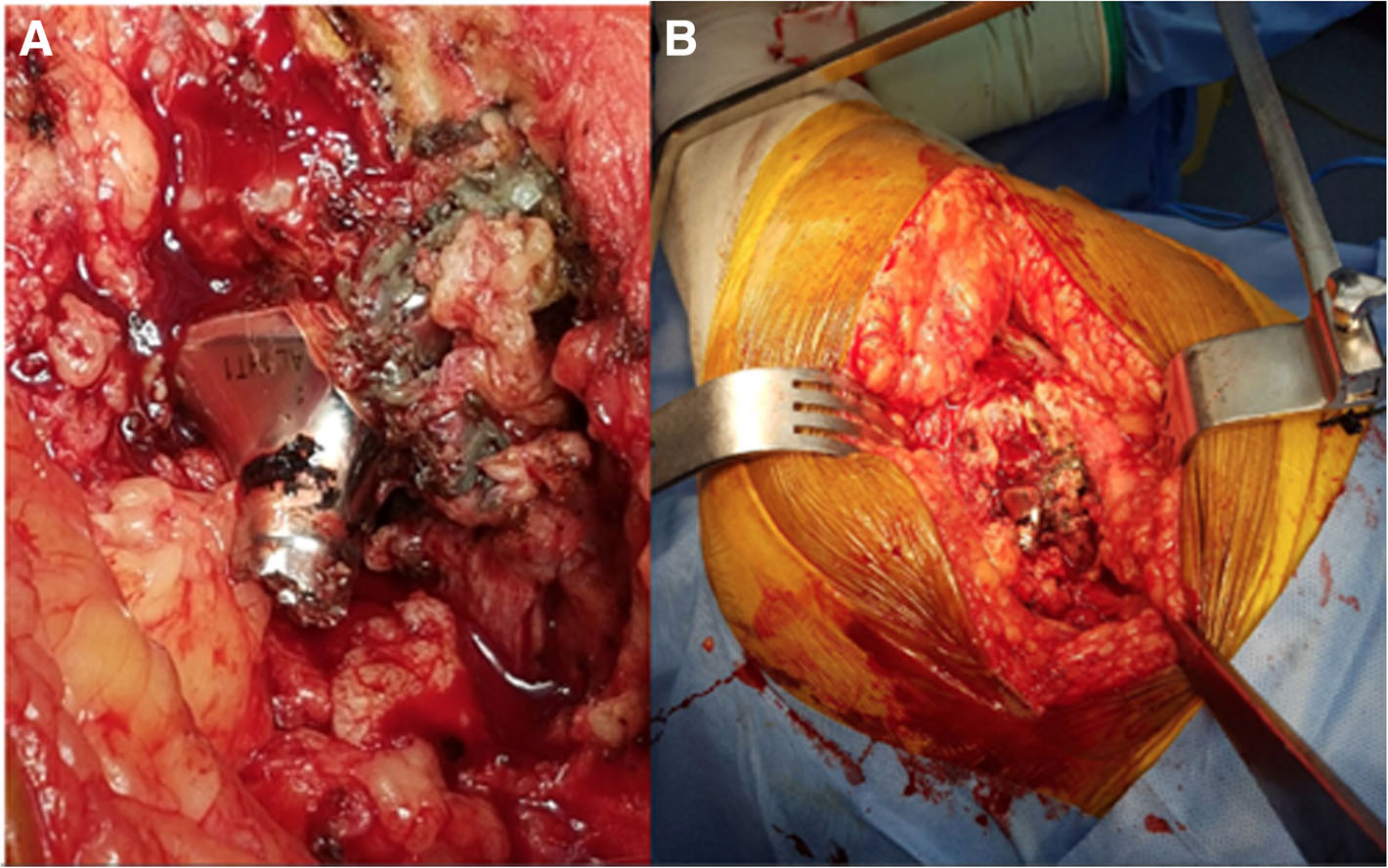
**a** Intra-operative photograph showing the broken trunnion with corrosion at the fracture site and some metallosis. **b** Close view

**Fig. 3:**
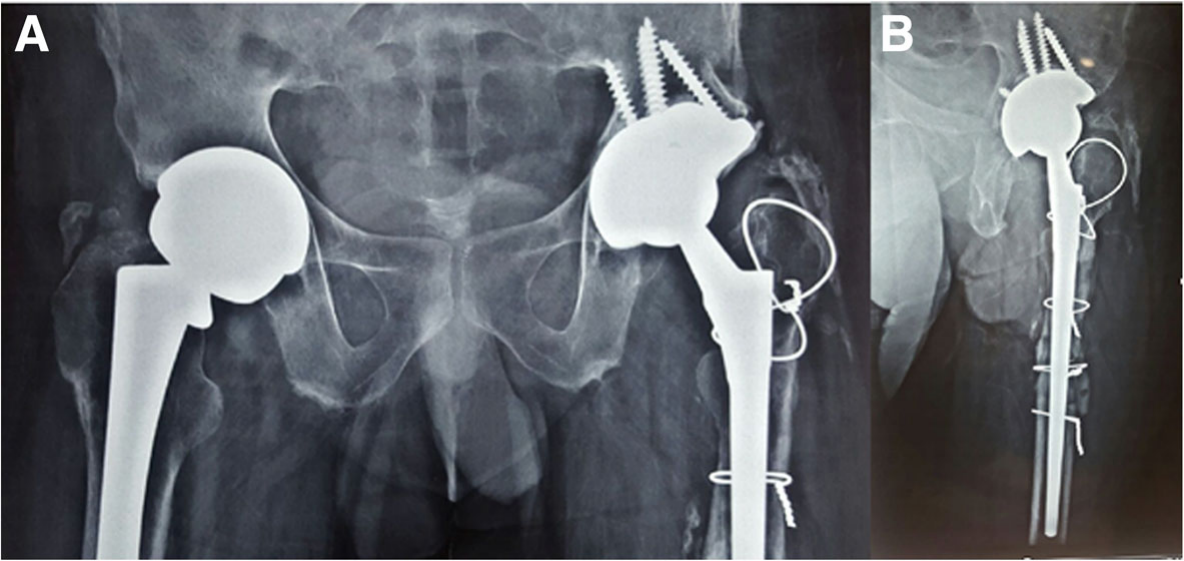
Radiographs at 6 weeks following surgery, showing trochanteric and femoral wiring for extended trochanteric osteotomy, and the revised stem and cup on the left hip. **a** Anteroposterior view. **b** Lateral view

**Fig. 4:**
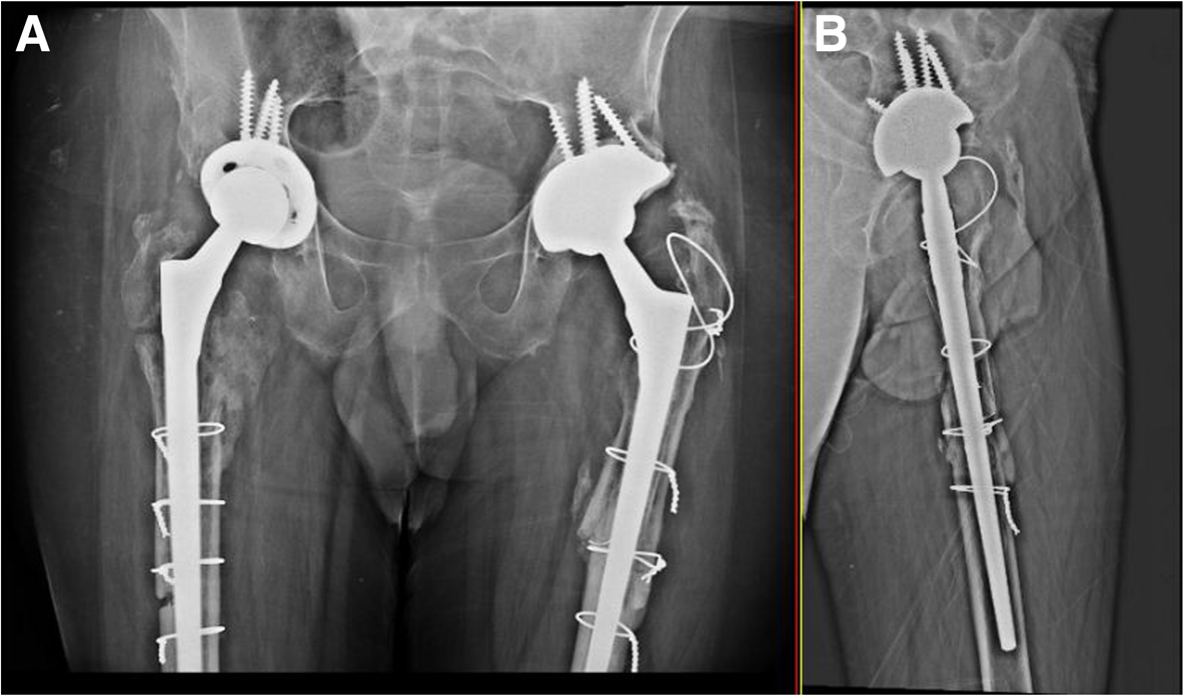
Radiographs at 3 years follow up. **a** Anteroposterior view. **b** Lateral view

He subsequently underwent revision left THA. Both the femoral and acetabular components were revised. Intraoperatively, we found evidence of fretting corrosion at the fracture site (Fig. 2a, b). The trunnion fracture was oblique in pattern and extended superolaterally to inferomedially, exactly at the head-neck articulation. It suggested a stress riser effect and cyclic fatigue failure. The proximal part of the broken trunnion was still attached to the femoral head. We also found evidence of localized metallosis and soft tissue reaction. The femoral component was extracted by means of an extended trochanteric osteotomy and revised to a long cementless Wagner SL stem (size 14, Zimmer, Warsaw, USA). The acetabular component was extracted using an explant device and was revised to an uncemented continuum multi-hole cup (size 54 mm, Zimmer, Warsaw, USA) along with a Delta ceramic head (size 36 mm; + 7), UHMWPE liner (54 mm/36 mm), and acetabular screws. In addition, the postero-superior defect of the acetabulum was reconstructed using a Tantalum augment (size 54; thickness 15 mm). Following surgery, the patient had an uneventful recovery. Post-operative radiographs showed satisfactory alignment of the components (Fig. 3a, b). He continued to have a good functional outcome after 3 years, and the radiographs at the last follow up were as shown in Fig. 4a, b.

## Discussion

Trunnionosis is defined as wear of the femoral head-neck interface and is increasingly recognized as a cause of failure and revision following THA. It can be asymptomatic, but sometimes manifests as localized metallosis, adverse local soft tissue reactions, and even catastrophic failure or fractures of the implants. The actual incidence of adverse reactions including metallosis far exceeds the revision rates. Some studies indicate that the revision rate following metal-on-metal THA ranges from 19 to 22% at 10 years, while that of metal-on-polyethylene THA ranges from 3 to 4% [5].

The AML stem was designed as early as 1979 by Lord *et al* [6]. The early design features included a straight, cobalt-chrome, extensively porous coated stem with distal diaphyseal fixation. The stem had an excellent track record with a survivorship of 98% at 20 years. The AML A plus stem in our case was specifically designed for the Asian population as the existing standard stems did not cater to the smaller sizes required in this group of patients. It has a smaller neck taper of 9/10, unlike the standard 12/14 taper, and was originally designed for the 22.5 mm diameter Charnley-type metal heads. Subsequently, 28 mm metal heads were also successfully implanted. In the early part of the twenty-first century, large metal heads were also implanted worldwide with this stem, given the popularity of large metal-on-metal articulations and the excellent track record of the AML stem. This was especially true for young active individuals in whom reducing the wear rate and risk of dislocation was important.

Large metal-on-metal articulations have gone out of favour since then, and have been recalled in view of adverse reports of proximal stress shielding and anterior thigh pain [7–10]. There were also issues regarding increased blood cobalt and chromium ion levels, nephrotoxicity, adverse reaction to metal debris (ARMD), pseudotumours and a probable carcinogenic effect due to chronic elevated blood metal ion levels. Gross trunnion failure (GTF) or dissociation at the femoral head-trunnion interface is also increasingly being recognized as a cause of failure and revision surgery [2, 11].

Fractures of the trunnion are rare. Unnanuntana *et al* reported two cases of trunnion fractures with AML A plus femoral components at 6 and 7 years post-operatively [12]. In both cases, a 28 mm metal head was used with + 6 neck offset. Peterson *et al* reported a case of trunnion fracture following revision THA with a metal-on-polyethylene bearing with a 32 mm (+ 10) head [2]. Banerjee *et al* in their series of cases with trunnion-related failure reported a case of trunnion fracture at the base of the skirted sleeve, following cemented THA using a 28 mm head [1].

In our case, the primary cause of the fracture was the imbalance caused by a large metal head on a small taper stem design, leading to increased bending moment at the trunnion. This aggravated the cantilever effect, causing subsequent cyclic fatigue failure. It was further accentuated by the high offset (+ 6 size head). Other contributory factors could include the young age and high activity levels of the patient, grooves on the trunnion design causing a stress riser and corrosion at the head-neck articulation site. Our case is unique since there are reports of trunnion fractures with AML stems and 28 mm metal heads, but to the best of our knowledge, none with large metal-on-metal articulations. AML A plus stem and ASR cups have since been recalled by the company, but there is a need to constantly monitor these patients for potential trunnion-related complications.

## Conclusions

The primary cause for the trunnion fracture in our case was the imbalance caused by a large metal head on a small taper stem which aggravated the canatilever effect. Patients who have undergone THA using a metal-on-metal articulation need to be monitored constantly for trunnion-related complications.

## Data Availability

Data sharing is not applicable to this article as no datasets were generated or analysed during the current study.

## Abbreviations

THA: Total Hip Arthroplasty
AML: Anatomic Medullary Locking
ASR: Articular Surface Replacement
UHMWPE: Ultra High Molecular Weight Poly Ethylene
